# Analyzing Katana referral hospital as a complex adaptive system: agents, interactions and adaptation to a changing environment

**DOI:** 10.1186/s13031-015-0046-5

**Published:** 2015-06-08

**Authors:** Hermès Karemere, Nathalie Ribesse, Bruno Marchal, Jean Macq

**Affiliations:** Université Catholique de Bukavu, Sud-Kivu, RD Congo; Université Catholique de Louvain, IRSS, Bruxelles, Belgique; Institute of Tropical Medicine, Antwerpen, Belgium

**Keywords:** Referral Hospital, Health care, Performance, Armed conflict, Complex adaptive system, South Kivu, Democratic Republic of Congo

## Abstract

This study deals with the adaptation of Katana referral hospital in Eastern Democratic Republic of Congo in a changing environment that is affected for more than a decade by intermittent armed conflicts. His objective is to generate theoretical proposals for addressing differently the analysis of hospitals governance in the aims to assess their performance and how to improve that performance. The methodology applied approach uses a case study using mixed methods ( qualitative and quantitative) for data collection. It uses (1) hospital data to measure the output of hospitals, (2) literature review to identify among others, events and interventions recorded in the history of hospital during the study period and (3) information from individual interviews to validate the interpretation of the results of the previous two sources of data and understand the responsiveness of management team referral hospital during times of change. The study brings four theoretical propositions: (1) Interaction between key agents is a positive force driving adaptation if the actors share a same vision, (2) The strength of the interaction between agents is largely based on the nature of institutional arrangements, which in turn are shaped by the actors themselves, (3) The owner and the management team play a decisive role in the implementation of effective institutional arrangements and establishment of positive interactions between agents, (4) The analysis of recipient population’s perception of health services provided allow to better tailor and adapt the health services offer to the population’s needs and expectations. Research shows that it isn’t enough just to provide support (financial and technical), to manage a hospital for operate and adapt to a changing environment but must still animate, considering that it is a complex adaptive system and that this animation is nothing other than the induction of a positive interaction between agents.

## Introduction

The Health zone is the operational health unit of the health system in the Democratic Republic of Congo (DRC), a pyramidal one which has three levels (central, intermediate and operational levels). The central level, the top of the pyramid, corresponds to the national Health Ministry. Its role is to develop policies, strategies and standards for the operationalization of the whole system. The intermediate or provincial level includes offices in charge of the organization of health care in the province. It provides technical support to the operational level in terms of training, monitoring and supervision. The Health Zone (HZ) is a set of health centers linked to a district hospital - called general referral hospital (GRH). So, the health district model, “Health Zone” in the DRC, is a common approach to organizing health services [1], including disadvantaged populations [2]. Within this type of system, the role played by the GRH is well established [3, 4]. Patients needing care exceeding the first line’s capabilities are directed to these referral hospitals [4]. Health centers are considered as deconcentrated units of the hospital. They are in charge of primary health care. They refer in case of need to the GRH, which is in charge of second line care and expected to give referral feed-back to the health centers. As such, the health zone is organized as a local health system. A secondary referral hospital normally lies in the provincial capital. The GRH is a faith-based hospital in some health zones. Faith-based hospitals provide a significant portion of healthcare in low-income countries [5] and they are often considered to perform better than public hospitals [6].

In this study, we focus on the Katana GRH, a faith-based hospital, which has been operating since 1928. Katana GRH is located in South Kivu province in eastern DRC. It has a maternity ward, a pediatrics department, an internal medicine department, a surgical ward, an intensive care unit and a medical imaging department, which provides ultrasound and endoscopy services. The hospital is connected with 17 health centers. In 2010, 52 % of patients which attended the outpatient department had been sent by health centers, as well as 75 % of patients in inpatient department [7]. The bed occupancy rate in 2010 was 78 %. There were 121 employees in 2010, including 7 medical doctors (general practitioners) and 59 nurses. Some nurses work as pharmacist, anesthesiologist, laboratory technician, radiologist, nutritionist, dentist and ophthalmologist. Roughly 19 % of nurses were trained on the spot and did not acquire formal nursing diploma [7–9]. Doctors and the nursing director are part of the health zone management team, in charge for the supervision of health centers and directed by the health zone’s head doctor. The hospital benefited from technical and financial support from the Belgian government for many years, even when instability, violence and war outbroke in the South Kivu province [10].

The study that we report in this paper is the last part of a larger study concerning the determinants of adaptation to a changing environment in several hospitals of Eastern DRC. In a previous case study in Ituri, we analyzed how Logo and Bunia GRH adapted to a new development aid programme. The study not only measured production of services, but also analyzed the interactions between agents [11]. In fact, three key agents were identified in Bunia and Logo: the hospital management team (comprised of the hospital’s medical director, the administrative manager, the nursing director, the medical chief of staff, and, in some cases, the Health Zone’s head doctor), the hospital staff (comprised of other doctors, nurses, administrators and support staff), and the owner (represented by the Provincial Medical Inspector for state hospitals, as in Bunia, or by the head of the “Bureau Diocésain des Oeuvres Médicales”, a diocesan medical office for faith-based hospitals, as in Logo). The analysis of the interaction between these 3 hospital agents led to the following theoretical propositions:

1. The nature of the interactions between the three key hospital agents (management team, hospital staff and hospital owner) contributes to the hospital’s stability or fragility in the face of factors as divergent as war or new health programs.
2. The interactions between these agents are framed in pre-existing institutional arrangements at the hospital (known as the requirements and house rules, both formal and informal, anchored in its recent and distant history and culture), while the strength and type of these interactions depend on information (including communication), finance and human resources management.

From a complexity theory perspective, a hospital consists of many different actors. The multiple interactions between the various players leads to a degree of self-organization and collective behaviour, which in turn, may influence individual behavior and other elements of the system [12-14]. Furthermore, hospitals are open systems, and in constant interaction with their environment, while past events and decisions may have an influence on today’s situation. In short, hospitals can be considered to be complex adaptive systems [15]. Adaptation leads to change, the dynamics of which are influenced by characteristics of the organization, such as production monitoring by professionals (owners of expertise and knowledge), the presence of groups with multiple and often diverging interests, and by power systems [16].

In the present study, we took Katana GRH as the case to test and refine the propositions that resulted from the Bunia and Logo GRH case studies [17, 18]. We analyzed the changes at the Katana hospital by analyzing the interaction of the hospital as a system and its environment.

Many authors define an agent as an actor (or a group made up of actors) who interact with others in a social space (in this case, a hospital). These interactions are meant to strengthen one’s position by simultaneously mobilizing cooperation and confronting opposite interests [19-21], in the case of a hospital management team with the aim of maintaining the hospital’s main resources. We identified three main agents who interact at the hospital: the management team (the MT, driven by both a managerial and medical/clinical logic), the hospital staff (HS; with a mainly medical logic), and the hospital owner (HO, who has a managerial logic, and in case of the “Bureau Diocésain des Oeuvres Médicales” (BDOM), perhaps a religious motivation). The production of healthcare by the hospital depends on the interactions between these different agents. Within this configuration of agents, each agent is inextricably and recursively characterized by his/her mental and cognitive structures (worldview, knowledge, beliefs, intentions and plans); the resources (economical, social and biological) that he/she owns or can mobilize and that co-defines his/her position at the hospital; and, finally, his/her dispositions [19]. Any change in one of these three areas can lead to changes in the two others, as well as in the relationships that the agent has with his/her own environment and coworkers.

## Methods

### Using the case-study approach to refining an initial theory

In order to refine the propositions that resulted from our earlier studies, we used the case study design. We analyzed three historical periods at the Katana GRH between 1990 and 2010 that were considered as major turning points with respect to the hospital’s activities and evolution. Each period was considered “a case” that was explored through the lenses of complex adaptive systems theory.

### Collection and data analysis

1. Document review

Hospital annual reports, training workshop reports, management or steering committee meeting reports, reports from the board of directors’ missions and from partner organizations’ technical assistants, as well as from executives at the intermediate level, were analyzed to identify critical events and interactions during the hospital’s lifetime. The identified elements were validated through interviews with key informants.

2) Hospital health care production data

Data were collected retrospectively from the hospital’s health information system. We analyzed the evolution of the annual care production at Katana GRH, differentiating between “Consultations - Admissions”, “Surgery”, and “Maternity”. During the war time, the monitoring of hospital services provision and performance were partly neglected. The available data was used for the present study. More specifically, we used the following eight indicators: the new consultation rate, the hospital admission rate, the major surgical intervention rate, the volume of appendectomies, the volume of blood transfusions, the number of ultrasounds, the volume of hospital deliveries and the caesarean section rate. Graphing the data helped not only to assess the evolution of the production, but also to differentiate stable from unstable periods.

3) Key informant survey

We carried out a survey in which 14 key informants were involved. They were selected on the basis of the following criteria: to be actively involved in the hospital’s management and to have spent at least 5 years working at Katana GRH. The informants included district medical officers, hospital medical directors, medical chiefs of staff, nursing directors, administrative managers, head nurses, mid-level executives, hospital owner representatives, technical assistants working in the donor funded development programme, and other actors working for non-governmental organizations who provided local support for the Katana hospital management team during the study period (1990-2010). The survey took place from July to December 2011 with the help of an open-ended questionnaire, which was created after having analyzed the evolution of the hospital indicators, as well as the events and interventions that took place at Katana GRH. The survey aimed at validating key events and interventions identified during the document review, identifying other relevant events, understanding the reaction of the management team to these events and interventions, and obtaining an interpretation of the observed variability in the hospital’s production of care by key informants. The questionnaire was administered either in person (n = 8) or over the internet (n = 6) as a self-administered survey, completed by guidance over the phone when necessary. For analysis purposes, the data were recorded in Excel tables, respecting the anonymity of respondents. Key informants’ answers were labeled by number (IC1, IC2, IC3…, and IC14) to better analyze agents interactions.

**Fig. 1:**
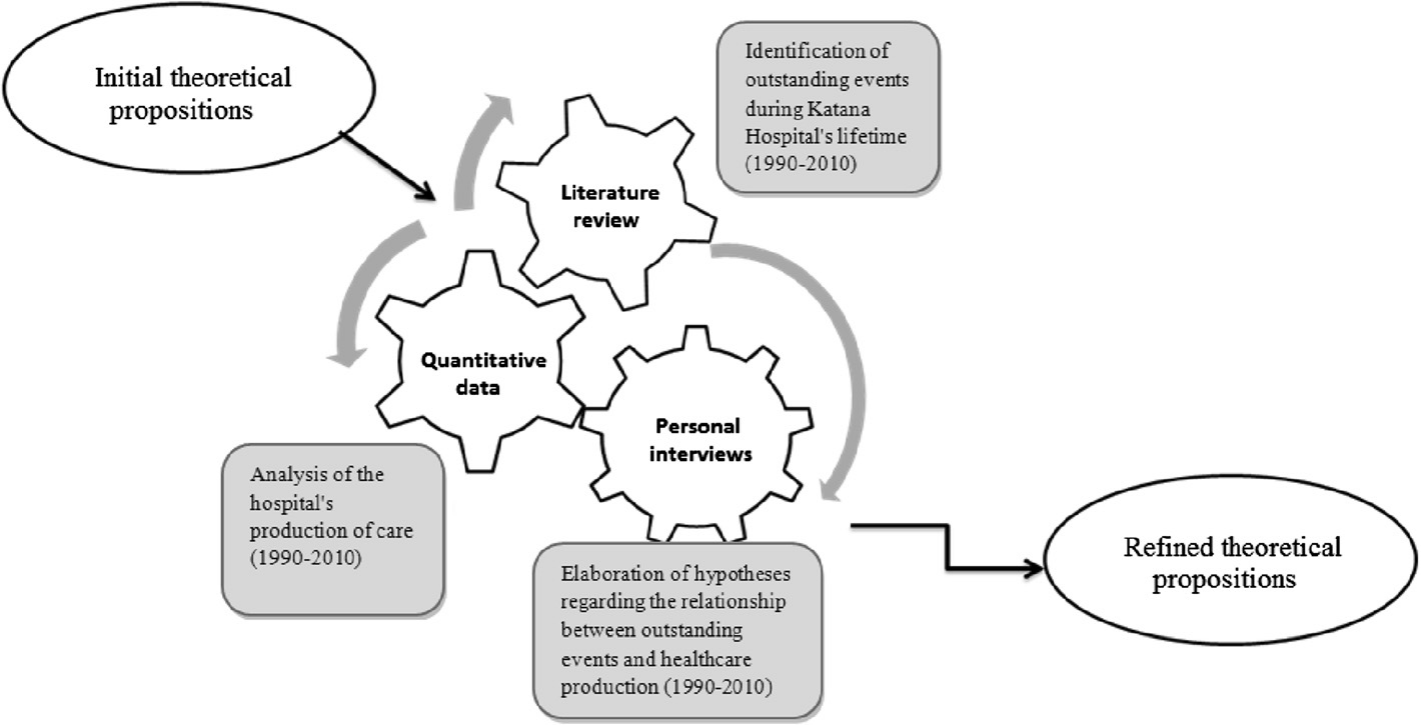
Methodological approach

The initial analysis focused on key elements of the theoretical propositions, such as context, the three groups of key hospital agents (owner, management team and hospital staff), and hospital organization in terms of information, communication, finance and human resources management. Secondly, we explored whether the events in the environment affected the hospital production and searched for the mechanisms that allowed to facilitate the hospitals’ adaptation. The confrontation and triangulation of the three sources of information allowed us to test and refine the initial theoretical propositions (see Fig. 1).

#### Ethical considerations

Before its implementation, the research protocol was submitted to the DRC Ministry of Health for review and was discussed by the *Groupe de recherche en appui à la politique sur la mise en œuvre de l’agenda pour l’efficacité de l’aide à la suite des déclarations de Paris et d’Accra (*GRAP-PA), a forum of Belgian researchers. Since this study was part of a doctoral thesis project, the protocol was reviewed by the Doctoral Committee of the Catholic University of Louvain. This study didn’t require any other ethical approval. Verbal consent was asked to the people interviewed.

## Results

### Overview of the critical events and interventions of the last 20 years

Upon completion of the document review and the interviews, 35 out of 116 events and interventions occurring over a period of 20 years as key events were identified.

**Fig. 2:**
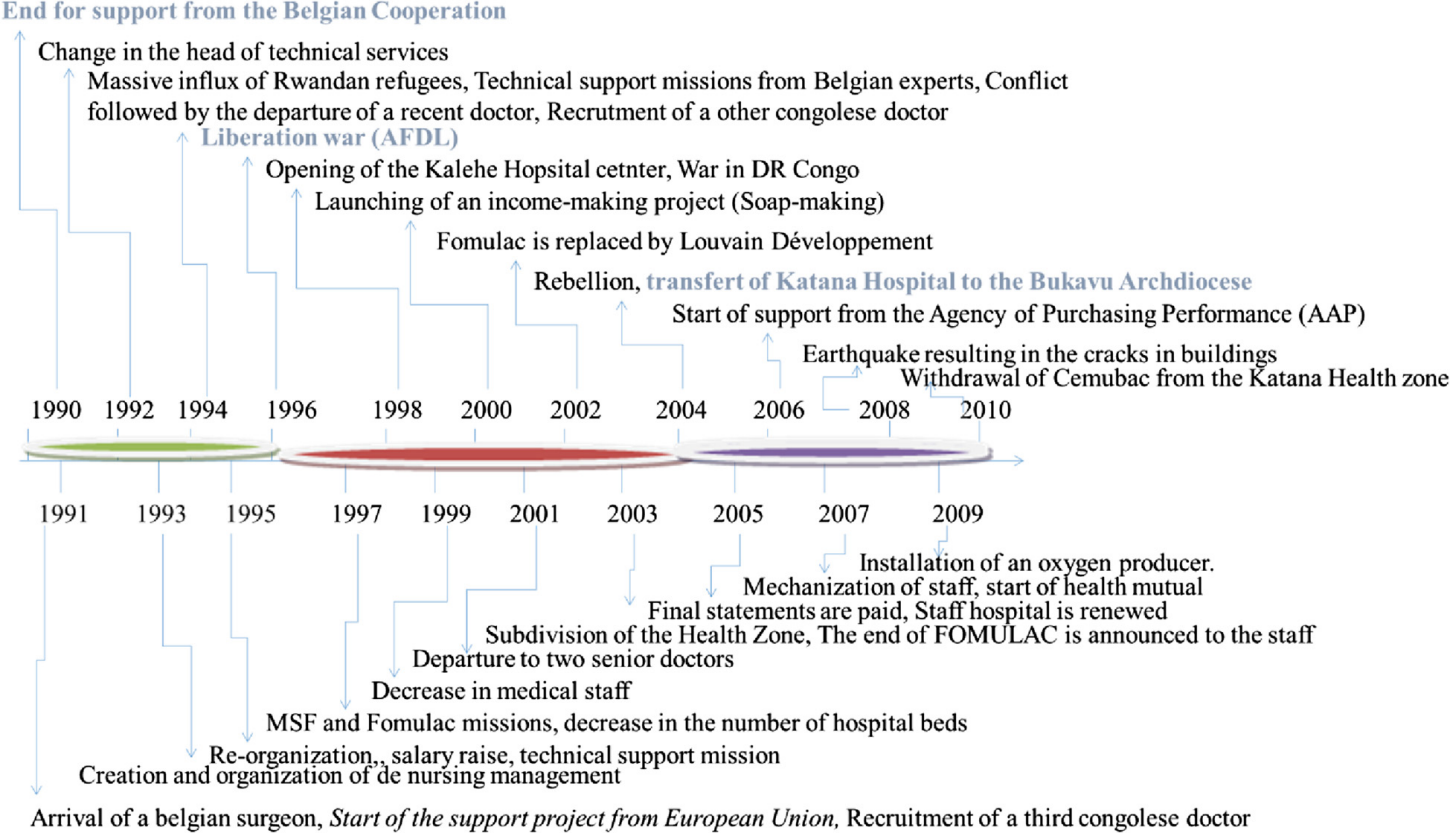
Study periods and the historical changes occurring at Katana Hospital

Our analysis was focus on three key moments that were identified by 86 % of the respondents as having a major impact on Katana GRH’s activities. This includes the collapse of the Belgo-Congolese Cooperation in 1990, the outbreak of the “liberation war” in 1996 and the change of the hospital owner in 2004. We defined three periods based on these turning points (see Fig. 2). As such, the first period goes from 1990 to 1995, the second period from 1996 to 2003, and the last from 2004 to 2010. As mentioned in the Methods section, we took these periods as cases.

### The period 1990–1995: the collapse of Belgo-Congolese cooperation

The main changes noted were the collapse of the Belgo-Congolese Cooperation in 1990, the implementation of a Survival Program supported by the European Union in the Katana Health Zone in 1991 and the outbreak of the Rwandan war in 1994, which was followed by a massive influx of Rwandan refugees and humanitarian help in the South Kivu province.

The main changes in actors during this period were the departure of expatriate doctors and the Belgian medical director of the hospital in the wake of the rupture between the Belgian and the Congolese states and the withdrawal of the Belgian NGO FOMULAC *(Fondation Médicale de l’Université de Louvain en Afrique Centrale),* which was in charge of the hospital. A Congolese medical director was appointed in 1990 and doctors were recruited for the medical staff, which included a surgeon expatriate.

#### Key informants’ interpretations about the collapse of the Belgo-Congolese cooperation

The collapse of the Belgo-Congolese cooperation and the departure of Belgian collaborators were seen as an abrupt abandonment of the Katana GRH (IC2, IC3, IC6, IC8, IC10, and IC13). According to the interviewees, this resulted in a considerable reduction of funding (IC3, IC5, IC10, IC12, IC13), and was detrimental to the hospital activities, staff salary payments (resulting in a staff strike in 1991) and medical supplies (stock depletion in 1991).

The hospital’s funding was highly dependent on Belgian subsidies, which covered over 80 % of the budget (IC8). The hospital management team had not anticipated a sudden withdrawal of the main funder and had no alternative funding strategy (IC2, IC7). After the Belgian withdrawal, some of the Congolese staff were prepared to take over the management role, while others left the hospital. The doctors who finally took over the management of the hospital consequently were overloaded with work (IC7, IC8). The management team obtained temporary salary subsidies from the Congolese government, allowing the hiring of an expatriate surgeon in 1991 and the end of the staff strike.

The respondents’ viewpoints regarding the hospital’s performance after the departure of the Belgians were mixed: according to some actors, performance was maintained (IC7, IC9), while other actors claim that performance declined (IC13, IC14). Contact with the Belgian NGO, FOMULAC, was maintained, which enabled a distance technical support for the management team (IC8).

#### Dynamics of interactions between agents

To compensate the departure of the expatriate doctors, the management team hired a Congolese doctor. The new doctor had never worked at the Katana GRH, and as a result, he was not familiar with Katana GRH’s organizational culture and not sure on how to manage his responsibilities. In the end, conflict broke out and the management team decided to terminate his contract. At the same time, a request by staff members for a salary increase ended in a full-blown strike. As already mentioned, the management team obtained salary subsidies from the Congolese state, and interacted with the owner, who helped obtain financial support from the European Union to run the hospital and the Health Zone, which ended the strike.

#### Changes in production of services (see Fig. 3a)

**Fig. 3:**
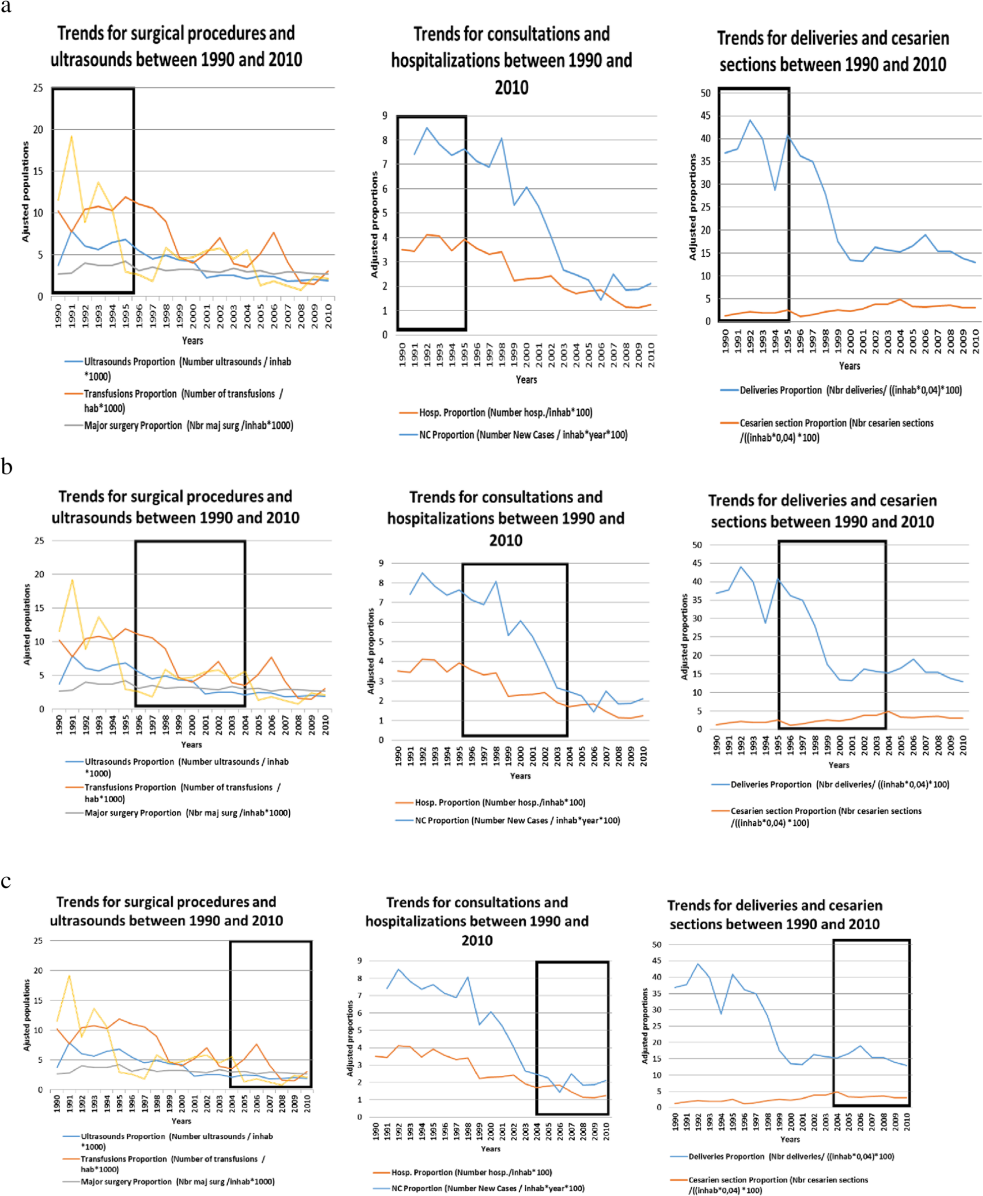
**a** Trend of consultations, hospitalizations, deliveries, cesarean sections, surgical procedures and ultrasounds between 1990 and 1995. **b** Trend of consultations, hospitalizations, deliveries, cesarean sections, surgical procedures and ultrasounds between 1996 and 2003. **c** Trend of consultations, hospitalizations, deliveries, cesarean sections, surgical procedures and ultrasounds between 2004 and 2010

Our analysis of the data show that this period is marked by a relative stability in terms of production of services, except for deliveries (a temporary decline) and appendectomies (a significant decline).

### The period 1996–2003 : the outbreak of the first “liberation war”

The outbreak of the first “liberation war” in 1996 led to the sudden departure of the doctors in charge of the hospital. In response, an Emergency Management Committee was put into place, which was dissolved 7 months later when the doctors who fled the war returned. The medical staff responsibilities were redistributed accordingly.

In 1997, members of the FOMULAC board of directors formulated recommendations for the hospital’s reorganization during a visit to Katana, which included recruiting doctors native to the region in order to ensure continuity and staff turnover decrease.

The year of 2001 was characterized by the successive resignations of the two first Congolese medical directors (who were also respectively head of the Health Zone and medical hospital director); they were the oldest members of the hospital medical staff. They were replaced that same year by staff doctors, who were introduced to the administrative tasks.

Besides the first “liberation war”, events occurred in this period are: the outbreak of the first liberation war in the DRC in 1996, the opening of the Kalehe hospital center about 21 km from Katana GRH and the renewal of violence with the outbreak of the second “liberation war” in DRC in 1998. There was also the subdivision of the Katana Health Zone into four new Health Zones, with the setting up of new hospitals in 2003. Finally, the Ministry of Health introduced a new policy on supervision of health centers to improve the accessibility and quality of health care.

#### Changes in the production of services (see Fig. 3b)

During this period saw a significant decrease in “primary care” activities or activities that should have taken place in more peripheral structures around Katana GRH (outpatient consultations, uncomplicated deliveries). The number of cesarean sections and major surgical interventions remained stable.

#### Key informants’ interpretations about the outbreak of the war

To ensure their safety, experienced doctors and hospital administrators left Katana at the outbreak of the war (IC2, IC12), even if one respondent argued that their departure was somewhat unnecessary as they were not personally threatened (IC6). An Emergency Management Committee was set up for these war conditions, and the management team was in close contact with the hospital owner. They discussed the arrival of a MSF surgeon and introduced the idea of recruiting native doctors (IC2), as well as the need to prepare younger doctors for higher responsibilities (IC10). Being protected by the population, the hospital was not rampaged by the rebels nor soldiers. It was, however, seriously destabilized, as were many other structures within the region (IC3). The remaining younger doctors quickly took over the management (IC4) at the expense of a slightly tarnished reputation of the hospital, which resulted from diminished confidence of the population in the medical staff (IC5).

Following this change of hands, dynamic of interactions between the agents changed. A sense of disorientation led to reduced coordination between hospital units and the hospital’s management team. Health worker morale suffered (IC8, IC10). A respondent noted that the departure of the senior doctors foreshadowed what could become the Katana GRH if their departure had been permanent and not adequately prepared for (IC8). The arrival of a surgical team from an MSF mission a few months later mitigated the impact of this rupture on the patient care, particularly in the surgical unit (IC13).

### The period 2004–2010: the transfer of the hospital to Bukavu archdiocese

This period is characterized by a change in hospital ownership; in 2004, FOMULAC handed over Katana GRH to the Bukavu Archdiocese, which incorporated the hospital into its faith-based health care system and changed the hospital management team. The announcement of the plan to transfer Katana GRH to the Bukavu Archdiocese led to the resignation of numerous healthcare and administrative workers. During the following year, the management team underwent major changes, as members were frequently transferred within the faith-based network.

This played out against the background of contextual changes that include the insurrectional war in 2004, a cooperation intervention implementing a system of performance financing through an agency of services purchase (“Agency for Purchasing Performance”) since 2006, the damage due to the earthquake in 2008 and the global financial crisis in 2009, which reduced the support of the hospital’s partners.

During this period, production of services remained stable but at a low level (with the exception of transfusions), and no improvements were seen (see Fig. 3c).

#### Key informants’ interpretations of ceding Katana GRH to Bukavu archdiocese

The ceding of Katana GRH by FOMULAC to the Bukavu Archdiocese was seen as a sign of loss of faith in the hospital and as the abandonment of loyal and dedicated staff, which created much frustration (IC3). A staff crisis resulted, which eventually ended with the payment of end-of-contract settlements (IC4, IC13). The hospital’s staff had to re-apply for their posts and was interviewed by the staff of the Archdiocese. The subsequent recruitment was perceived as based on subjective grounds and compared to a “witch hunt” (IC5, IC10). The providers and even the population immediately expressed reluctance towards the BDOM and had a very bleak view of the ever increasing presence of the Bukavu Catholic church when it came to the hospital’s management (IC5). They feared the instauration of an new management system, which was criticized in other hospitals under the BDOM’s responsibility, especially with regard to staff management, a domain where the rules were not clear (IC5, IC6). Staff feared change (IC6) and more salary cuts, which would risk to lead to decreased motivation and care quality (IC9). It was therefore not in the hospital’s best interest to suddenly bring down an entire system that was functioning well in the eyes of numerous agents (IC11, IC14), even if a need to replace certain incompetent or elderly staff members was acknowledged (IC12). Respondents felt that the transition could have been achieved differently. The end-of-contract payments, for instance, could have been made more rapidly by the FOMULAC. This missed opportunity was perceived to be the result of unnecessary haste to finalize the transfer (IC12, IC14).

Katana GRH had a hard time adapting to the departure of the FOMULAC during the first year (IC2, IC6, IC9, IC11), especially because of the new management system and because of the loss of support, including medical supplies (IC6, IC9, IC11). Whereas the hospital management team in the past could rely on a comprehensive support system, the new management system organized by the Archdiocese was based on the payment of the actual cost of care. Being unprepared for this change of funding modality, the functioning of the hospital was disrupted and access to care became difficult for the population (IC2, IC3, IC5, IC12). The new system also resulted in a significant downsizing, which included ending staff benefits, such as healthcare coverage for staff family members. Under the new system, staff were encouraged to join a community health insurance. At the same time, the management team attempted to form new partnerships with other technical and financial stakeholders (IC5). The BDOM’s efforts somehow stabilized the situation through intensified supervisions, continuous staff training, and advocacy for additional funding for the hospital from other hospital partners (IC13).

#### Dynamics of interactions between actors

The transfer of Katana GRH to the Bukavu Archdiocese was preceded by a number of departures: the resignation of senior doctors in 2001; the departure of the Missionary Sisters for Africa in 2003, who were there since 1938 in playing a primary role at the maternity ward, the pharmacy, the internal medicine department for women, and the hospital’s social services department; the resignation of multiple staff members, including some executives. The transfer was accompanied by a radical change at all levels (Management Team, Hospital Staff, and Hospital Owner). Only the Hospital Staff kept a few members of the preexisting team, which faced with changing values and institutional arrangements. In addition to establishing a health care insurance policy, the new owner created several new partnerships with the hospital.

## Discussion

This study aimed at testing and refining two theoretical propositions on how the management team and the key actors in a hospital adapt to a changing environment in a volatile setting such as the South Kivu province of the DRC. The first proposition stipulated that the stability or fragility of a hospital exposed to external factors such as war depends on the interaction between three key hospital actors, i.e. the management team, the hospital staff and the hospital owner. The second proposition stipulates that the interaction between these actors is the result of pre-existing institutional arrangements and that the strength and type of these arrangements depend on established management mechanisms of (1) information, including communications, (2) funding, and (3) human resources.

### Methodological considerations

Our main method consisted of identifying key events within and outside of the hospital, linking these in a timeline and analyzing how the interaction between 3 groups of actors shaped this interaction. We encountered some limitations, including the major difficult to have accurate financial hospital data and detailed information on the behavior of each agent within this complicated context. We only limited our interview to people considered as central players, and we did not interview patients nor the general population, for they were not directly involved in the management of Katana GRH. Nonetheless, we recognize the major role that patients play in the hospital’s performance. We tried to minimize recall bias (especially for people who were interviewed many years after their departure from Katana) by triangulating the information collected from interviews with information from the document review. This triangulation helped reduce bias and increased the reliability and validity of the study [22, 23]. The interviews had brought more precise and detailed information than the auto administered questionnaires sent by email to the far key informants. Additional information was obtained by phone to clarify certain answers from the auto administered questionnaires allowing to minimize the gap between informant’s answers from the two data collection methods. Likewise, the evolution of the quantitative data expressed in ratio (more plausible) was “stackable” to the data expressed in whole number for the considered indicators.

### Positive interactions between agents as a determinant for adaptation to change

During the first phase we discussed, the end of the Belgo-Congolese cooperation in 1990 was a key contextual factor that led to the departure of a number of expatriate staff and the end of Belgian funding for the hospital. As a result, drug and supplies stocks were depleted and the staff went on strike. One would therefore expect to see a drop in production following this event [24]. However, adaptive actions were undertaken. Firstly, pro-active leadership by the management team led staff salary payments from the government, ended the staff strike. Secondly, new funding for the hospital was obtained through involvement of the hospital owner. Thirdly, the recruitment of a Belgian surgeon was initiated by both the management team and the hospital owner. As a result of this positive interaction, the hospital managed to continue providing care and even its improvement it as shown in Fig. 3c. The management team, supported by the hospital owner, played a key role in facilitating the hospital’s adaptation to change, mobilizing other agents (the hospital staff and contextual actors) in the process.

### Institutional arrangements as a lever for adapting to change

When the Katana GHR was transferred to the Bukavu Archdiocese in 2004, the stable relations between the agents were severely disrupted. Some of the institutional arrangements were re-evaluated and modifications were made. When the Bukavu Archdiocese took over Katana GRH, it brought in new partners, including the Agency for Purchasing Performance (APP), which in turn led to new funding modalities. Although we did not assess the specific impact of the APP, the Archdiocese institutionalized management networking practices which it has developed for all its diocesan hospitals. It also changed the way of managing human resources: staff members floated from one diocesan hospital to another, including the managers. Access to free healthcare for staff members and their families was replaced by a mandatory membership to a local health insurance scheme. Perhaps more importantly, unlike FOMULAC, which acted via the management team, the new owner operated mainly through the hospital staff. This was both an advantage and a disadvantage for the management team; while their role was in a sense now supported by other staff members, the management team felt as though their initiative had been undermined and the team cohesion diminished. The frequent change of staff, especially of hospital management team members, furthermore deprived the hospital of the opportunity of consolidating and stabilizing interactions between the hospital’s agents.

### The role played by expatriate doctors, multifaceted national and expatriate technical assistance and humanitarian funding in the hospital’s adaptation to change

Expatriate doctors and several national and international experts intervened in Katana GRH to address the population health needs, in particular surgery needs. These interventions had an impact on population’s perception on the quality of services provided. Despite the territorial division of the health zone and the opening of new hospitals, the evolution of surgeries was constant during the period of observation, reflected an effective response of surgery department to the patients’ needs and expectations. In other departments, in particular in outpatient service and maternity, the production of services decreased. Population’s perception of health care provided, strongly influenced in the past by the presence of expatriate doctors in surgical department, constitutes an important measure to adapt health services offer to the population’s need and expectations.

#### New theoretical propositions

Based on the analysis of the 3 periods at Katana GRH, we refine the initial theoretical propositions as follows:

(1) Interaction between key agents is a positive force driving adaptation if the actors share a same vision. (2) The strength of the interaction between agents is largely based on the nature of institutional arrangements, which in turn are shaped by the actors themselves.(3) The owner and the management team play a decisive role in the implementation of effective institutional arrangements and establishment of positive interactions between agents. (4) The analysis of recipient population’s perception of health services provided allow to better tailor and adapt the health services offer to the population’s needs and expectations.

## Conclusion

How hospital management teams adapt to a changing environment in an unstable context relies on their management vision and on the nature of interactions with three other main agents, the hospital staff, the owner of hospital and the population. The institutional arrangements that are in place, which depend on the owner’s attitude and on the degree of leadership by the management team, determine the strength and type of interaction, but they are shaped inevitably by the relations between groups of actors and their power positions, including population’ power. National or international expertise is required to support all administrative activities, both managerial and clinical, that take place in a hospital within an unfavorable context. The interaction between agents should be strong in order to avoid having the expert replace the management team and in order to ensure that the expert provides true participatory support. For a hospital to run smoothly, it is not enough to lead, manage, and evaluate it; one must also (and perhaps especially) animate it, both at the managerial level and the clinical level. This animation is nothing more than the initiation of a positive interaction between agents through a same vision.
